# Interaction With the Extracellular Matrix Triggers Calcium Signaling in *Trypanosoma cruzi* Prior to Cell Invasion

**DOI:** 10.3389/fcimb.2021.731372

**Published:** 2021-10-04

**Authors:** Nubia Carolina Manchola Varón, Guilherme Rodrigo R. M. dos Santos, Walter Colli, Maria Julia M. Alves

**Affiliations:** ^1^ Laboratory of Biochemistry of Parasites, Department of Biochemistry, Institute of Chemistry, University of São Paulo, São Paulo, Brazil; ^2^ Department of Clinical Pathology, State University of Campinas, Campinas, São Paulo, Brazil

**Keywords:** *Trypanosoma cruzi*, calcium signaling, extracellular matrix, Chagas disease, mitochondria

## Abstract

*Trypanosoma cruzi*, the etiological agent of Chagas disease in humans, infects a wide variety of vertebrates. Trypomastigotes, the parasite infective forms, invade mammalian cells by a still poorly understood mechanism. Adhesion of tissue culture- derived trypomastigotes to the extracellular matrix (ECM) prior to cell invasion has been shown to be a relevant part of the process. Changes in phosphorylation, S-nitrosylation, and nitration levels of proteins, in the late phase of the interaction (2 h), leading to the reprogramming of both trypomastigotes metabolism and the DNA binding profile of modified histones, were described by our group. Here, the involvement of calcium signaling at a very early phase of parasite interaction with ECM is described. Increments in the intracellular calcium concentrations during trypomastigotes-ECM interaction depends on the Ca^2+^ uptake from the extracellular medium, since it is inhibited by EGTA or Nifedipine, an inhibitor of the L-type voltage gated Ca^2+^ channels and sphingosine-dependent plasma membrane Ca^2+^ channel, but not by Vanadate, an inhibitor of the plasma membrane Ca^2+^-ATPase. Furthermore, Nifedipine inhibits the invasion of host cells by tissue culture- derived trypomastigotes in a dose-dependent manner, reaching 95% inhibition at 100 µM Nifedipine. These data indicate the importance of both Ca^2+^ uptake from the medium and parasite-ECM interaction for host-cell invasion. Previous treatment of ECM with protease abolishes the Ca^2+^ uptake, further reinforcing the possibility that these events may be connected. The mitochondrion plays a relevant role in Ca^2+^ homeostasis in trypomastigotes during their interaction with ECM, as shown by the increment of the intracellular Ca^2+^ concentration in the presence of Antimycin A, in contrast to other calcium homeostasis disruptors, such as Cyclopiazonic acid for endoplasmic reticulum and Bafilomycin A for acidocalcisome. Total phosphatase activity in the parasite decreases in the presence of Nifedipine, EGTA, and Okadaic acid, implying a role of calcium in the phosphorylation level of proteins that are interacting with the ECM in tissue culture- derived trypomastigotes. In summary, we describe here the increment of Ca^2+^ at an early phase of the trypomastigotes interaction with ECM, implicating both nifedipine-sensitive Ca^2+^ channels in the influx of Ca^2+^ and the mitochondrion as the relevant organelle in Ca^2+^ homeostasis. The data unravel a complex sequence of events prior to host cell invasion itself.

## Introduction

*Trypanosoma cruzi*, the etiological agent of Chagas disease, infects approximately 7 million people, most of them living in the American continent (World Health Organization, 2020). Replicative and infective forms are present throughout the *T. cruzi* life cycle, which alternates between insect vectors from the reduviidae family and mammalian hosts. Briefly, mammals are infected by *T. cruzi* metacyclic trypomastigotes forms present in the feces and urine of contaminated insects during a blood meal and, closing the life cycle in nature, the invertebrate vectors are infected by trypomastigotes (blood trypomastigotes) during a blood meal in contaminated mammals. Epimastigotes and amastigotes are the replicative forms of the parasite in the digestive tract of the insect and in the cytoplasm of the mammalian cells, respectively, followed by their differentiation into trypomastigotes. Additionally, amastigotes released by the rupture of cells are able to infected other mammalian cells. Congenital infection, blood transfusion, or organ transplants are other forms of *T. cruzi* transmission (World Health Organization, 2020).

*T. cruzi* trypomastigotes invade both phagocytic and non-phagocytic mammalian cells (Tanowitz et al., 1975) by a poorly understood complex process. *In vitro* studies have highlighted the interaction of tissue culture- derived trypomastigotes with the extracellular matrix (ECM) as an important step of the invasion process. These trypomastigotes bind to distinct ECM components, such as laminin (Giordano et al., 1999), heparan sulfate (Calvet et al., 2003), collagen (Velge et al., 1988), fibronectin (Calvet et al., 2004), thrombospondin (Ulrich et al., 2002; Johnson et al., 2012; Nde et al., 2012), galectin-3 (Da Silva et al., 2017), as well as TGF-β (Nde et al., 2012; Silva et al, 2019). Additionally, extracellular vesicles secreted by the parasite (Gonçalves et al., 1991), soluble factors, and distinct proteases (Santana et al., 1997; Scharfstein et al., 2000; Motta et al., 2012; Watanabe Costa et al., 2016) acting on the host extracellular matrix influence the invasion process. Moreover, remodeling of the ECM by *T. cruzi*, with an increment in the synthesis of collagen IV, fibronectin, and laminin (Cardenas et al., 2010; Coelho et al., 2018), was described in cardiac cells. Increase in thrombospondin-1 in the case of human colon epithelial cells (Suman et al., 2018) was associated with fibrosis, which occurs in chronic Chagas disease patients (Magalhães-Santos et al., 2004; Garzoni et al., 2008; Nájera et al., 2021). Notwithstanding, biochemical events triggered by ECM on trypomastigotes during their mutual interaction are less explored (Nde et al., 2012). Coexistence of tissue culture- derived trypomastigotes with ECM for 2 h (late phase) leads to large cellular responses, with changes in the phosphorylation (Mattos et al., 2019), S-nitrosylation (Mule et al., 2021), and nitration levels of proteins (Magalhães et al., 2020), suggesting a pre-adaptation of the parasite to its new intracellular environment. Reprogramming of the *T. cruzi* metabolism upon incubation with the ECM, involving kinases and phosphatases, was also described (Mattos et al., 2019), as well as global changes in the binding pattern of nitrated proteins to DNA, indicating changes in the chromatin structure, possibly affecting nuclear functions (Magalhães et al., 2020). Of note, nitrated histones, with the identification of the modified residue in histone H2B (Y29) by mass spectroscopy, were identified for the first time in trypomastigotes (Magalhães et al., 2020). Although the signaling pathways were not determined, the data show that the interaction of tissue culture- derived trypomastigotes with the ECM for a 2-h period activates distinct signaling responses before the parasite reaches the intracellular milieu of the host cells. However, the early events triggered by the interaction of *T. cruzi* trypomastigotes with the ECM have not been explored yet.

Calcium signaling pathways control many diverse processes in all eukaryotic cells and, particularly, Ca^2+^ is essential for the invasion of host cells by *T. cruzi* trypomastigotes, even though differences between metacyclic and tissue culture-derived trypomastigotes in the invasion process were described (Moreno et al., 1994; Yakubu et al., 1994; Ruiz et al., 1998). Although the mechanism is not fully understood, increment in the intracellular Ca^2+^ concentration in trypomastigotes, as well as in host cells, occurs at early stages of the invasion process (Tardieux et al., 1994; Moreno and Docampo, 2003; Chiurillo et al., 2019). The increment appears to be necessary in processes such as the fusion of the plasma membrane with the lysosome during invasion (Burleigh and Andrews, 1998). Acidocalcisomes, which are important Ca^2+^ stores in trypanosomatids, were implicated in this Ca^2+^ increment, since the inositol 1,4,5- triphosphate receptor (IP3R) localizes to the acidocalcisomes (Lander et al., 2016) and ablation of the IP3R gene inhibits host cells invasion by trypomastigotes (Chiurillo et al., 2020). In addition to acidocalcisomes, the single mitochondrion of *T. cruzi*, the endoplasmic reticulum, and the plasma membrane are involved in Ca^2+^ homeostasis and signaling (Lu et al., 1998; Lander et al., 2018), with different Ca^2+^ transporters implicated (Ramakrishnan et al., 2018). L-type voltage gated Ca^2+^ channels (VGCCs) (Prole and Taylor, 2011) and Ca^2+^ ATPase (PMCA) (Docampo and Huang, 2015) are responsible for Ca^2+^ uptake and extrusion, respectively, in the plasma membrane. Recently, a sphingosine–dependent Ca^2+^ channel and voltage independent was described for the Ca^2+^ uptake in the plasma membrane of *T. cruzi* (Rodriguez-Duran et al., 2019) and *Leishmania* (Benaim et al., 2013). Although this channel resembles the human L-type VGCCs, it differs in its activation by sphingosine and can be explored as a target for drug development (Ramakrishnan et al., 2018; Benaim et al., 2020). Considering the main Ca^2+^ storage organelles, a calcium uniporter complex (MCUC) and a Ca^2+^/H^+^ exchanger for Ca^2+^ uptake and Ca^2+^ release, respectively, are present in the inner mitochondrial membrane; a Ca^2+^-ATPase for Ca^2+^ uptake, in addition to the mentioned IP3R for calcium efflux is present in acidocalcisomes. Although a sarcoplasmic-endoplasmic reticulum-type Ca^2+^ ATPase (SERCA) for the Ca^2+^ uptake was described, no Ca^2+^ release mechanism was identified, since the IP3 receptor is absent in the endoplasmic reticulum (Chiurillo et al., 2020).

As pointed out above, we identified relevant changes in several metabolic pathways of *T. cruzi* trypomastigotes after a 2-h interaction with the extracellular matrix. Modifications in the phosphorylation, nitration, and S-nitrosylation levels, reprogramming of metabolism, as well as alterations in the chromatin structure, possibly affecting nuclear functions, strongly suggest a broad effect of parasite adhesion to the ECM, a step that precedes the invasion itself. Here, an increment in the Ca^2+^ concentrations at an early phase of the tissue culture-derived trypomastigotes interaction with the ECM is described, implicating nifedipine- sensitive Ca^2+^ channels present at the plasma membrane. Additionally, inhibition of the Ca^2+^ uptake by this channel impairs the trypomastigote invasion of mammalian cells. Data also support the conclusion that the sole mitochondrion of the parasite is the major organelle in this aspect of Ca^2+^ signaling.

## Materials And Methods

### Incubation of Tissue Culture-Derived Trypomastigotes With the Extracellular Matrix (ECM) (MTy)

Cultured-derived trypomastigotes from *T. cruzi* (*Y* strain) were obtained by the infection of epithelial rhesus monkey cells (LLC-MK_2_) as described (Andrews and Colli, 1982). The parasites released on the fifth day after infection of LLC-MK_2_ were collected and purified on DEAE-Cellulose chromatography as described (de Sousa, 1983; Cruz-Saavedra et al., 2017). Then, 5 x 10^8^ parasites in 5 ml of Modified Eagle’s Medium (MEM) supplemented with 2% FBS (MEM-2%FBS) were mixed with up to 100 µL (containing between 10–45 µg of protein, as quantified by Bradford assay) of commercial ECM (Geltrex™ LDEV-Free Reduced Growth Factor Basement Membrane Matrix, Invitrogen, lots 1790948, 2030111 or 2007114) and incubated at 37°C and 5% CO_2_ for different periods of time (between 0–20 min) according to the experiment, as described by Mattos et al. (2019) or with ECM elements (5 µg of collagen-1, heparan sulfate, thrombospondin, laminin-111, fibronectin). After the incubation, the parasites were centrifuged at 4,000 x g for 10 min and the pellets stored at -80°C until use (MTy samples). As a control, tissue culture-derived trypomastigotes were submitted to the same experimental conditions, except for the absence of the ECM (Ty samples). ECM-protease treated was obtained by incubating 40 µg of ECM with 5 mg/mL of proteases (P0652 Sigma) for 1 h at 35°C, followed by the addition of 50 µl of protease inhibitor cocktail (P8465 Sigma). Collagen-treated collagenase was obtained by incubating 5 mg of Collagen I with collagenase (C6885 Sigma) as described.

### Immunofluorescence

Tissue culture-derived trypomastigotes (1 x 10^8^) were incubated in MEM-2%FBS in the presence or absence of 100 µM Nifedipine (481981 CalbioChem) for 2 h at 37°C and 5% CO_2_. After incubation, the parasites were washed with PBS and fixed in 2% paraformaldehyde (PFA) for 2 h, washed, and incubated with anti-Tubulin antibody (1:100) (Sigma-Aldrich Cat# T8328, RRID: AB_1844090) in PBS-0.1% BSA. After an overnight incubation at 4°C, the samples were washed three times with PBS and incubated with Alexa-Fluor anti-mouse 488 secondary antibody (1:5,000), following the instructions of the manufacturer. The images were taken on an ExiBlue™ camera (Qimaging^®^) coupled to a Nikon Eclipse E 600 optical microscope and deconvoluted using the ImageJ 1.x software as described by Schneider et al. (2012). The exposition time was the same for each sample, in order to compare the parasite morphology.

To verify the Nifedipine (Nif) effect during the infection process, tissue culture-derived trypomastigotes were incubated in the presence of different Nif concentrations (0–100 µM, freshly prepared) for 2 h in MEM–2%FBS at 37°C and 5% CO_2_, as above, centrifuged, resuspended in 500 µL of MEM-2% SFB, and employed to infect LLCMK_2_ cells. LLCMK_2_ cells (1 x 10^5^ cells/well in 24-well plates) were previously cultivated in MEM-10%FBS at 37°C and 5% CO_2_. After 24 h, each well containing the LLCMK_2_ cells was infected with 1 x 10^7^ trypomastigotes previously incubated in the presence or absence of Nif. After 2 h, the wells were washed 4x with PBS, followed by incubation in MEM-2% FBS at 37°C and 5% CO_2_ for 48 h. After an extensive wash with PBS, the cells were fixed by the addition of 2% PFA for 2 h. The wells were then washed 2x with PBS, treated with 0.01% TritonX-100 for 5 min, followed by the addition of 0.5 µg/mL of DAPI for staining the nucleus of the cells. The images were acquired in a DMi8 Leica microscope. To determine the rate of infection, the nucleus of LLCMK_2_ and parasite cells, corresponding to 400 host cells, were counted using the Fiji Image J cell counter plugin. The pictures are representative of three independent experiments.

### Immunoblotting

Tissue culture-derived trypomastigotes, 1 x 10^8^ per sample in MEM–2%FBS, were incubated (or not) with 48 µg of ECM for 0, 5, or 10 min in the presence of 1 mM EGTA, 10 nM Okadaic acid (Sigma, Cat# O9381), or 4 µM Nifedipine in the same conditions described above. After incubation, the parasites were washed twice with PBS, resuspended in 500 µl of PBS supplemented with 20 µl of protease inhibitor cocktail (Sigma, Cat# P8465) and 20 µl of phosphatase inhibitor cocktail (Sigma, Cat# P2850). The cells were disrupted by mechanical force (Sonication 3 times at 20 power) and centrifuged at 5,000 g for 10 min. Proteins were separated by the SDS-polyacrylamide gel electrophoresis (12% SDS-PAGE), followed by the immunoblotting procedures, as described by Mattos et al. (2019), using rabbit anti-phosphorylated proteins (Pan) (Thermo Fisher Scientific Cat# 61-8300, RRID : AB_2533941), home-made mouse monoclonal anti-paraflagellar rod antibody (Anti -PFR) (1:10,000), mouse monoclonal anti-Collagen TI (1:1,000) (Sigma-Aldrich Cat# C2456, RRID : AB_476836), and the corresponding HRP-conjugated secondary antibodies. Due to the presence of ECM in the experiments involving trypomastigotes incubated with ECM, samples corresponding to the parasite number (1 x 10^6^ parasites/lane), and not to the protein content, were loaded. Densitometry of phosphorylated proteins from the immunoblottings is expressed in Arbitrary Units normalized by the paraflagellar rod protein content using a home-made monoclonal antibody (Mattos et al., 2019). Three independent experiments were performed and the statistical significance calculated (1way ANOVA) P< 0.05.

### Intracellular Free Calcium Quantification

Trypomastigote forms purified by the DEAE-Cellulose chromatography as described above were washed twice with cold buffer A (116 mM NaCl, 5.4 mM KCl, 0.8 mM MgSO_4_, 5.5 mM D-glucose, 50 mM HEPES, pH 7.2). The parasites were resuspended to a final concentration of 1 x 10^9^ cells per mL in buffer A supplemented with 1.5% sucrose, 3uM Fluo-4AM fluorescent dye (Sigma, Cat# F14201) and 0.02% of Pluronic F-127 and then incubated for 1 h at 37°C under constant agitation and protected from light. After incubation, the parasites were washed three times with cold buffer A. The maximum fluorescence was determined by measuring the fluorescence of 50 µL (5 x 10^7^ parasites per measure) at λ ex: 495/em:520 nm in the presence of 2 mM CaCl_2_ in buffer A (at 37°C) after the addition of 0.03% Triton X-100, and the minimum fluorescence was determined by adding EGTA (100 mM) after 0.03% Triton X-100 [the concentration was calculated by the CaEGTA v1.3 (Bers et al., 2010) calculator from maxchelator.stanford.edu]. The presence of the dye was checked after the experiments by observation at the fluorescence microscopy. Intracellular free calcium levels [Ca^2+^]_i_ were determined by the following equation, where F is the AU of fluorescence and F_min_ and F_max_ correspond to the minimum and maximum F values, respectively:


$${\left[\text{C}{\text{a}}^{2+}\right]}_{i}=Kd*\left\{\frac{\left(F-F_{min}\right)}{\left(F_{max}-F\right)}\right\},Kd:345\;nM\;Fluo4\;AM$$

In order to determine whether the increase of intracellular free calcium levels was due to the extracellular calcium entry or release of calcium from any organelle, measurements were performed in the presence or absence of ECM and in 2 mM CaCl_2_-containing buffer A or 1 mM EGTA-containing buffer A. According to the experiment, different amounts of ECM were employed (between 0 and 40 µg/mL). ECM aliquots were maintained at -80°C and kept at 4°C before use in the experiments. The fluorescence changes were monitored in a Hitachi 7100 fluorescence spectrophotometer.

In order to disrupt the calcium flow through the parasite plasma membrane, 1 mM Vanadate (Van) or 4 µM Nifedipine (Nif) (freshly prepared) were added. To estimate the free intracellular calcium release from the mitochondria, acidocalcisome, and endoplasmic reticulum, 0.3 µg/mL of Antimycin A (AA) (Sigma, Cat# A8674), 1 µM Bafilomycin A (Baf) (MedChemExpress, Cat# HY-100558), and 0.3 µM Cyclopiazonic acid (CPA) (Sigma, Cat# C1530) were added, respectively, to trypomastigotes in the presence or absence of ECM. The data represent 5, 4 or 3 independent experiments for Baf, AA and CPA, respectively and the statistical significance calculated (1way ANOVA) P< 0.05.

### Measurement of Phosphatase Activity

Tissue culture- derived trypomastigotes (1 x 10^8^) were incubated (or not) with 40 µg of ECM for 10 min at 37°C in the presence of 1 mM EGTA, 4 µM Nifedipine, or 10 nM Okadaic acid (O9381 Sigma) (negative control), a specific inhibitor of serine/threonine phosphatases. The samples were centrifuged, washed two times with PBS, and resuspended in 500 µl of PBS supplemented with 50 µl of protease inhibitor cocktail (P8465 Sigma). The cells were disrupted by mechanical force (Sonication 3 times at 20 power) and the protein content of the samples was quantified by the Bradford assay. All the samples were kept on ice until the phosphatase assay. The phosphatase assay was performed as previously described (Meyer-Fernandes et al., 1999). Briefly, 1 mg/mL of the total extract was added to the reaction mixture [10 mM p-nitrophenyl phosphate (PNPP) (P4744 Sigma), 50 mM Tris-HCl pH 7.2] and incubated at 30°C for different periods of time (0, 10, 20, 30, 40, 60 min). The reaction was stopped by the addition of 1 mL of 1 N NaOH and the p-nitrophenol (PNP) produced was measured at 425 nm, using the attenuation coefficient of 1.8 x 10^4^ M^-1^cm^-1^. Three independent experiments were performed and the statistical significance calculated (One-way ANOVA) P < 0.05.

## Results

### Intracellular Free Calcium Concentrations Increase in Tissue Culture-Derived Trypomastigotes After Extracellular Matrix (ECM) Addition

**Figure 1:**
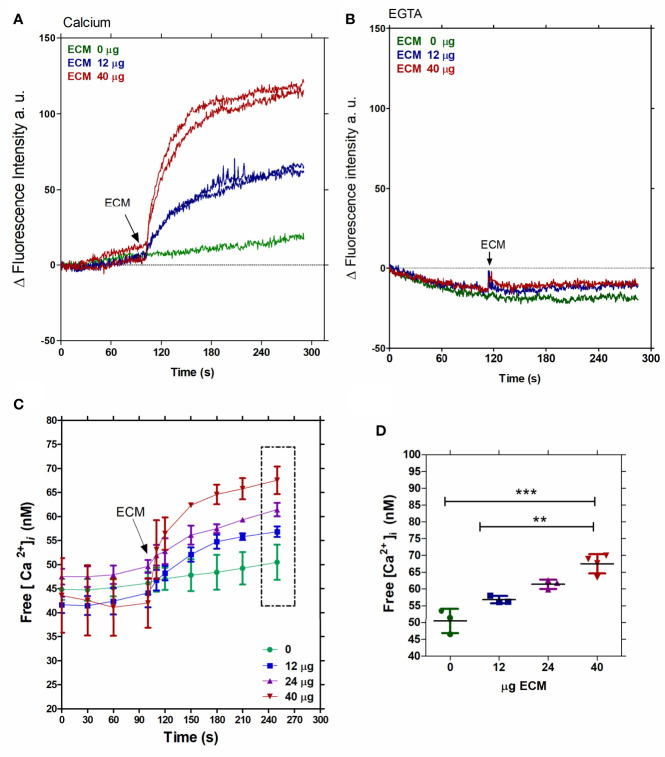
Free intracellular calcium increases in *T. cruzi* trypomastigotes upon interaction with the ECM. Fluo-4-AM-loaded tissue culture-derived trypomastigotes were employed. **(A)** Fluorescence intensity measured in the presence of two ECM concentrations (12 or 40 µg) and 2 mM extracellular Ca^2+^. **(B)** Fluorescence intensity measured in the presence of two ECM concentrations (12 or 40 µg) and 1 mM EGTA. **(C)** Increment of free intracellular [Ca^2+^] in response to the ECM concentrations (0, 12, 24, or 40 µg). **(D)** Dose-dependent free intracellular [Ca^2+^] and ECM concentration after 2.5 min of incubation, from **Figure 1C**. Statistical significance was P < 0.05, Tukey test ANOVA. **Figures 1A, B** are representative of three independent experiments, using three different commercial samples of ECM. **P < 0. 01, ***P < 0.001.

To investigate the role of the ECM on calcium signaling in *T. cruzi*, (5 x 10^7^ parasites per measure) Fluo4-loaded tissue culture- derived trypomastigotes (**Supplementary Figure 1- Video**) were first incubated with different ECM concentrations (0, 12, 24, and 40 µg) in order to establish a dose-dependent curve (**Figures 1C, D**). A rapid Ca^2+^ uptake was observed with a plateau reached in less than 2 min. At 2 min of incubation, the intracellular Ca^2+^ ([Ca^2+^] _i_) increased from 50 nM to 65 nM, in a manner dependent on the amount of the ECM (**Figure 1D**). The dependence on the extracellular Ca^2+^ was verified by the incubation of Fluo4-loaded trypomastigotes in a Ca^2+^-rich medium (2 mM CaCl_2_) (**Figure 1A**) or in a Ca^2+^ free extracellular medium (1 mM EGTA) (**Figure 1B**) after the addition of 12 or 40 µg of ECM. The uptake of Ca^2+^ by trypomastigotes was observed only when extracellular calcium was present (**Figure 1A**).

### Extracellular Calcium Uptake by Trypomastigotes Through Nifedipine-Sensitive Calcium Channels

**Figure 2:**
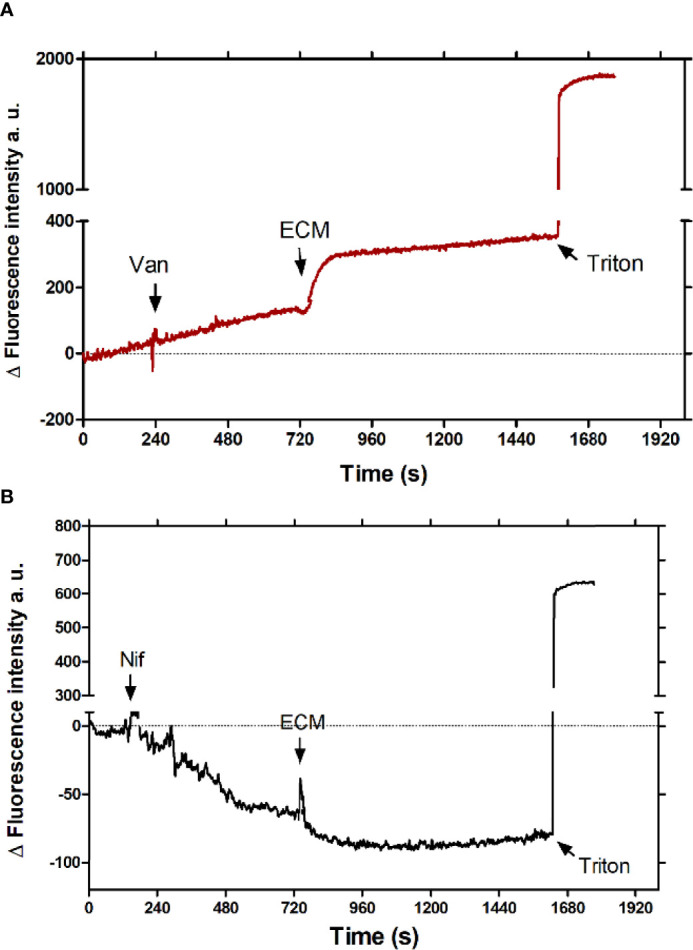
Effect of Ca^2+^ channel inhibitors in the intracellular Ca^2+^ concentration of tissue culture-derived trypomastigotes upon interaction with the ECM. **(A)** 1 mM Vanadate (Van) (red line) or **(B)** 4 µM Nifedipine (Nif) (black line) were added to 5 x 10^7^ Fluo-4-loaded trypomastigotes, followed by the addition of 24 µg of ECM after the signal stabilization. 0.03% Triton-X100 was added at the end of experiment to determine the maximum fluorescence value. The graph is representative of three independent experiments.

To identify the plasma membrane calcium channels involved in the tissue culture-derived trypomastigotes response to the ECM, Ca^2+^ uptake was measured in the presence of Vanadate, an inhibitor of the plasma membrane Ca^2+^ATPase, and Nifedipine, a 1,4-dihydropyridine, a well characterized inhibitor of L-type voltage gate Ca^2+^ channels (L-type VGCC) in humans, which inhibits the sphingosine-dependent plasma membrane Ca^2+^ channels described in trypanosomatids (Benaim et al., 1991; Rodriguez-Duran et al., 2019). As shown in **Figure 2**, the addition of 4 µM Nifedipine (Nif) totally blocked the extracellular Ca^2+^ uptake by Fluo-4-loaded trypomastigotes incubated with the ECM (**Figure 2B**), whereas 1 mM Vanadate (Van) showed no effect (**Figure 2A**). The data indicate that the increase in the intracellular free Ca^2+^ concentration in trypomastigotes observed during the ECM stimulus occurs *via* Nifedipine – sensitive calcium channels.

**Figure 3:**
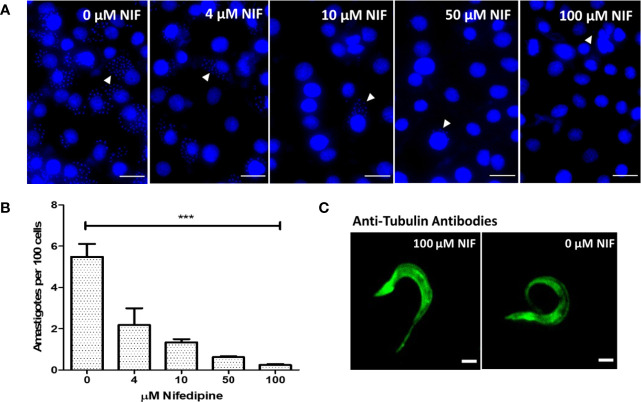
Extracellular Ca^2+^ uptake through Nifedipine- sensitive calcium channels is essential for tissue culture-derived trypomastigotes invasion. **(A)** Epithelial cells were incubated with trypomastigotes in the presence of 0, 4, 10, 50, and 100 µM Nifedipine (Nif) for 2 h and the infection analyzed 48 h later by the presence of intracellular *T. cruzi* amastigotes. White arrows indicate the amastigotes stained kinetoplasts; white bars represent 500 µm. **(B)** Quantification of 400 host cell infection from 3A. Statistical significance when compared with (0) (One-way ANOVA) P < 0.05. **(C)** Trypomastigotes incubated with 0 or 100 µM Nifedipine for 2 h, fixed, and developed with anti-tubulin antibodies. White bars indicate 5 µm. ***P < 0.001.

The role of Nifedipine was then tested in the invasion of epithelial cultured cells by trypomastigotes, since the relevance of Ca^2+^ in the process is well established in the literature. Trypomastigotes were incubated with 0 (control), 4, 10, 50, and 100 µM Nifedipine for 2 h and the infection level determined by counting the number of intracellular amastigotes (**Figures 3A, B**). A dose-dependent inhibition was observed, with 58% at 4 µM and 95% at 100 µM, the lower and the higher concentrations employed. Of note, no changes in the morphology (**Figure 3C**) nor in the motility of trypomastigotes were noticed after the treatments. The data point out the essential role of the extracellular Ca^2+^ uptake through Nifedipine-sensitive calcium channels in the trypomastigote invasion of mammalian cells.

### Organelles Involved in Calcium Homeostasis During Trypomastigote-Extracellular Matrix (ECM) Interaction

**Figure 4:**
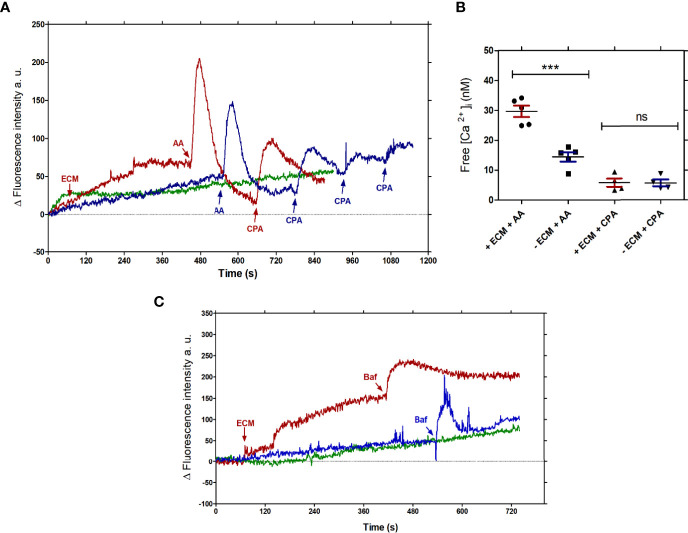
Organelles involved in the Ca^2+^ homeostasis in *T. cruzi* trypomastigotes upon interaction with the ECM. **(A)** Measurement of the intracellular free Ca^2+^ concentration in the presence of AA (0.3 µg/mL) or CPA (0.3 µM); Trypomastigotes + ECM (MTy, red line); Trypomastigotes (Ty, Blue line); basal signal from non-stimulated 5 x 10^7^ parasites (Green line). **(B)** Free calcium concentration measured in MTy incubated in the presence or absence of AA and CPA. The graphs are representative of five independent experiments, as shown in **Figure 4A**. The size of the peaks was estimated from the addition point to the maximum top. Statistical significance (One-way ANOVA) p < 0.01. **(C)** Measurement of the intracellular Ca^2+^ concentration of trypomastigotes in the presence or absence of Baf (1 µM); MTy (Red line); Ty (Blue line); basal signal from parasites not stimulated (Green line). The graph is representative of three independent experiments. MTy: 5 x 10^7^ trypomastigotes + 24 µg of ECM. ***P < 0.001; ns, no significant.

Intracellular Ca^2+^ homeostasis is rapidly reestablished by the organelles that are able to release or store calcium. Acidocalcisomes, mitochondria and endoplasmic reticulum are the main intracellular stores of Ca^2+^ in trypanosomatids, Their involvement in the uptake of Ca^2+^ increase in tissue culture- derived trypomastigotes incubated with ECM can be identified by the use of inhibitory conditions specific for each organelle. PMCA, a Ca^2+^- ATPase, similar to the plasma membrane-type Ca^2+^- ATPase, present in acidocalcisomes, is inhibited by bafilomycin A, a specific inhibitor of vacuolar-type H+ ATPases or by pH change of the organelles, e.g. by NH_4_Cl; endoplasmic reticulum has a sarcoplasmic-endoplasmic reticulum-type Ca^2+^- ATPase that is inhibited by cyclopiazonic acid; mitochondria have a MCU (Mitochondrial Calcium Uniporter) complex, that could be inhibited by analogues of ruthenium red and alternatively, its role can be shown by respiratory chain blockers, such as antimycin A or uncouplers of oxidative phosphorylation. In order to establish which intracelular organelles are involved in Ca^2+^ uptake, the amount of free intracellular Ca^2+^ was measured in Fluo-4-loaded tissue culture-derived trypomastigotes in the presence or absence of the ECM and specific Ca^2+^ homeostasis disruptors: (**Supplementary Figure 2**): Antimycin A (AA, 0.3 µg/mL) for mitochondria, Cyclopiazonic acid (CPA, 0.3 µM) for the endoplasmic reticulum (ER) (**Figures 4A, B**), and Bafilomycin A (Baf, 1 µM) for acidocalcisomes (**Figure 4C**). As shown in **Figure 4B**, the amount of free intracellular Ca^2+^ measured after AA treatment was higher than the sample without AA, and it is also higher than the control without the ECM (**Figure 4A**). No significant modifications were observed when CPA was added to trypomastigotes incubated or not with ECM (**Figures 4A, B**) in five independent experiments. These results show the relevance of mitochondria in the uptake of Ca^2+^, which is dependent on the membrane potential, disrupted by the AA inhibition of complex III from the electron chain. Although acidocalcisomes contain the IP3 receptor and are considered the main organelle involved in the calcium homeostasis (Huang and Docampo, 2020), 1 µM Bafilomycin A did not show significant differences between the sample in the presence or absence of ECM (free calcium concentration 11.17 ± 1.5 nM for Ty and 10.57 ± 0.98 nM for MTy) (**Figure 4C**). The data points out to the relevance of mitochondria in calcium homeostasis during the ECM-trypomastigote interaction.

### Phosphoprotein Profile of Trypomastigotes Upon Extracellular Matrix (ECM) Interaction

**Figure 5:**
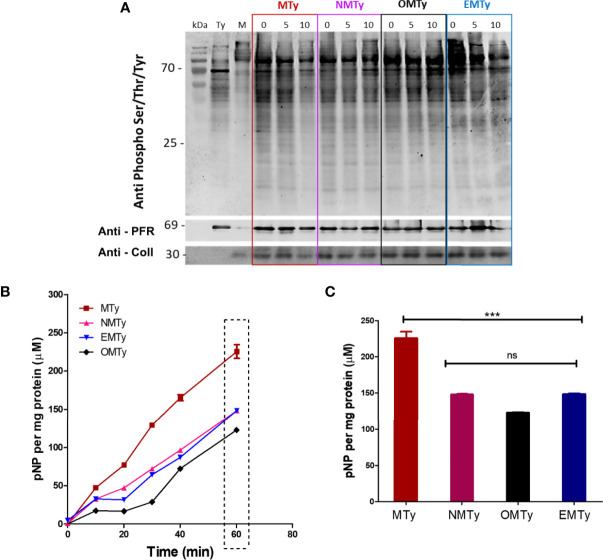
Phosphoprotein profile **(A)** and p-nitrophenyl phosphatase activity **(B, C)** from tissue culture-derived trypomastigotes upon interaction with ECM. Ty (trypomastigotes), MTy (+40 µg of ECM), NMTy (+40 µg of ECM and 4 µM Nifedipine), OMTy (+40 µg of ECM and 10 nM Okadaic acid), EMTy (+40 µg of ECM and 1 mM EGTA). **(A)** Immunoblot showing the phosphorylated protein profile of the samples incubated under the described conditions for different periods of time (0, 5, or 10 min). M corresponds to the control of the ECM (0.4 µg per lane); Anti-ColI (Collagen I) antibody indicates the presence of ECM in the sample (control); Anti-PFR (Paraflagellar Rod Protein) monoclonal antibody [control for the normalization of parasites loading (1 x 10^6^ parasites/lane)]. **(B)** Total p-nitro phosphatase activity of the MTy samples incubated with the drugs for 10 min. The production of pNP (p-Nitrophenol) was measured at different periods of time as indicated. The graph is representative of three independent experiments. **(C)** Bars representation of the square present in **(B)**, statistical significance (One-way ANOVA) P < 0.05. ***P < 0.001; ns, no significant.

Since phosphoproteomic analysis showed significant changes in the tissue culture-derived trypomastigotes incubated with ECM for 2 h (Mattos et al., 2019), the phosphoprotein profile was then analyzed in the early phase of the interaction by Western blot developed with anti-phospho-Ser/Thr/Tyr antibodies. To have a clue of the downstream signaling pathways, the phosphoprotein profile was analyzed by the incubation of 1 x 10^8^ tissue culture-derived trypomastigotes with 40 µg of ECM for 0, 5, or 10 min in the presence of 1 mM EGTA (EMTy), 10 nM Okadaic acid (OMTy), a cell permeant specific Ser/Thr phosphatase inhibitor, or 4 µM Nif (NMTy). Although the analysis of phosphorylated proteins was restricted to proteins below 70 kDa due to the presence of ECM in MTy (**Figure 5A**, lane M), the number of phosphorylated proteins was reduced in the trypomastigotes incubated with ECM for 10 min. Okadaic acid, EGTA, or Nif inhibit the dephosphorylation observed in MTy, showing the relevance of extracellular Ca^2+^ as an activator of phosphatases (**Figure 5A**). To confirm the results, the total p-nitro phosphatase activity was measured in cell extracts from the EMTy, OMTy, MTy, and NMTy samples from the 10-min incubation time (cf. **Figure 5A**). The phosphatase activity was measured for 60 min at 10-min intervals and the production of p-nitrophenol (pNP) was measured (**Figures 5B, C**). The presence of Nifedipine, EGTA, or Okadaic acid partially inhibited the phosphatase activity (~30%–35%), demonstrating the relevance of the Ca^2+^uptake for the total phosphatase activity. A slightly higher inhibition of enzyme activity was observed in the presence of Okadaic acid, an inhibitor of PP2A and PP1 protein phosphatases from *T. cruzi* (Szöör, 2010).

### Ca^2+^ Signaling in MTy Is Dependent on the Protein Components of the Extracellular Matrix (ECM)

**Figure 6:**
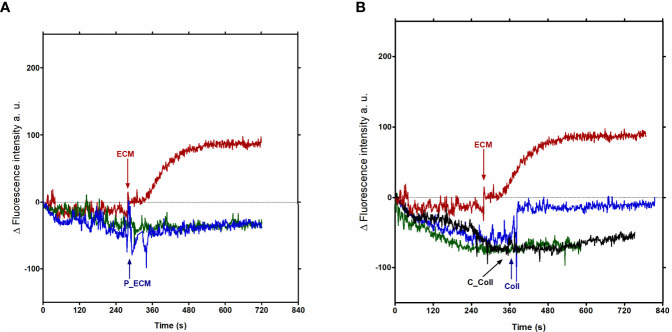
Protein fraction of ECM is implicated in the calcium signal in trypomastigotes from *T. cruzi*. Total ECM (40 µg) and Collagen I (5 µg)-derived calcium signal. **(A)** red line: Ca^2+^ concentrations measured in the presence of ECM; blue line: ECM previously treated with protease, followed by the addition of protease inhibitor (P-ECM); green line: Ty (trypomastigotes without ECM). **(B)** red line: Ca^2+^ concentrations measured in the presence of ECM; blue line: Ca^2+^ concentrations measured in the presence of 5 µg of Collagen I; black line: Ca^2+^ concentrations measured in the presence of 5 µg of Collagen I treated with collagenase (C-Coll). The graph is representative of two independent experiments.

Since the ECM components, including Collagen I (Velge et al., 1988), are implicated in the adhesion step of trypomastigotes when invading host mammalian cells, free intracellular calcium of Fluo-4-loaded trypomastigotes was measured in the presence of Collagen I (ColI). To verify the role of this ECM protein content in Ca^2+^ signaling, trypomastigotes were incubated with ECM or protease treated-ECM. ECM was pre-treated with protease, followed by the addition of protease inhibitors (P-ECM), as described in the Materials and Methods, before incubation with Fluo-4-loaded trypomastigotes. As shown, previous protease treatment abolished the extracellular Ca^2+^ uptake (**Figure 6A**). To confirm the relevance of proteins in the process, collagen was chosen to perform a similar experiment, since collagen was previously described as an adhesion protein in the invasion process of the parasite (Velge et al., 1988), and type I collagen is abundant in the ECM employed. Intracellular Ca^2+^ measured in trypomastigotes incubated with 5 µg of type I collagen previously treated with collagenase (C-Coll) is also abolished (**Figure 6B**), confirming the role of proteins. Although the increase in calcium measured in trypomastigotes incubated with 5 µg of collagen or 40 µg of ECM is similar (**Figure 6B**), the possible role of other ECM components in calcium signaling cannot be excluded. In addition to collagen-1, 5 μg of heparan sulfate also increases the intracellular calcium concentration in tissue culture- derived trypomastigotes (**Supplementary Figure 3**).

## Discussion

The invasion of host mammalian cells by *T. cruzi* trypomastigotes is a complex process which requires distinct surface molecules, and the activation of multiple signaling pathways in the parasites, as well as in the host cells (De Souza et al., 2010; Caradonna and Burleigh, 2011; Maeda et al., 2012; Giorgi and de Lederkremer, 2020; Nájera et al., 2021). Molecules from the parasite surface, such as members of the gp85/Transialidase glycoproteins coded by a multigenic family, bind to the ECM components, as a first step of the invasion process (Alves and Colli, 2008; De Souza et al., 2010; Nde et al., 2012), which triggers relevant changes in phosphorylation, S-nitrosylation, and nitration levels of proteins in *T. cruzi* trypomastigotes, Y strain, which elicit a reprogramming of the parasite metabolism (Mattos et al., 2019) or modification of the DNA binding profile of nitrated histones (Magalhães et al., 2020). However, the very early signaling events triggered in tissue culture-derived trypomastigotes by the ECM interaction were not explored.

Early studies showed Ca^2+^ increment during *T. cruzi* trypomastigote and extracellular amastigote or *Leishmania* sp. *amazonensis* amastigote interactions with host cells (Misra et al., 1991; Moreno et al., 1994; Yakubu et al., 1994; Lu et al., 1998; Hashimoto et al., 2014). Here, we show that the intracellular Ca^2+^ concentration increment in culture-derived trypomastigotes triggered by the interaction with the ECM is an early event, reaching a plateau in less than 2 minutes, which occurs by the uptake of extracellular Ca^2+^, since it is abolished by EGTA (**Figure 1**). Moreover, Ca^2+^ uptake by the plasma membrane is inhibited by Nifedipine, a dihydropiridine that blocks L-type voltage channels, and, as recently described, inhibits the sphingosine dependent plasma membrane Ca^2+^ channels in *T. cruzi* (Rodriguez-Duran et al., 2019) and *Leishmania* (Benaim et al., 1991; Benaim et al., 2013; Pinto-Martinez et al., 2018). In addition, Nifedipine drastically inhibits the invasion of host cells by *T. cruzi* trypomastigotes, reaching more than 90% inhibition at 100 µM of the drug (**Figure 3**) without affecting the parasite morphology or motility. These data confirm the essential role of Ca^2+^ in the invasion process described in the literature (Moreno et al., 1994) and establish a role of nifedipine-sensitive calcium channels in this uptake. Moreover, the results show that Ca^2+^ signaling starts at the adhesion step of the parasite to ECM, before the invasion of the host cell itself in spite that the downstream signaling, as well as the ECM component(s) that activate the calcium channel were not thoroughly identified.

In the particular case of the tissue culture-derived trypomastigotes-ECM interaction, the protein content of the ECM seems to be an important element for the induction of Ca^2+^ increase, since it is abolished by the previous treatment of the ECM with proteases (**Figure 6A**). The same conclusion resulted from similar experiments with Collagen I and with Collagen I previously treated with collagenase (**Figure 6B**). Collagen I, abundant in ECM isolated from epithelial cells employed here (Berkholtz et al., 2006), is one of the first ECM molecules described which trypomastigotes adhere to (Velge et al., 1988). However, the involvement of other ECM molecules in Ca^2+^ influx cannot be excluded.

The intracellular destination of the Ca^2+^ taken up during our experimental conditions was investigated. Mitochondria seem to be the main intracellular organelle responsible for buffering the free Ca^2+^ during the interaction of trypomastigotes with the ECM, as suggested by the Ca^2+^ increase in the presence of Antimycin A, a potent inhibitor of the mitochondrial electron transport chain (Grimmelikhuijzen and Slater, 1973; Stoppani et al., 1980) (**Figure 4**). The increment in Ca^2+^ uptake by the mitochondrial calcium uniporter during parasite-ECM interaction may stimulate ATP production through the activation of pyruvate dehydrogenase by the calcium-sensitive pyruvate dehydrogenase phosphatase (MCU-ATP-synthase megacomplex), as described previously (Lander et al., 2018). Interestingly, the direct interaction of MCU with subunit c of the ATP Synthase in the megacomplex, coupling the ADP and Pi transport and ATP synthesis, is present in trypanosomatids, as well as in humans (Huang and Docampo, 2020), thus providing energy for the subsequent steps of the invasion process. In contrast to the mitochondria, acidocalcisomes and endoplasmic reticulum seem to not play a prominent role in regulating the calcium concentration in our experimental conditions, as suggested by the data using 1 µM Bafilomycin A, a vacuolar H^+^ ATPase inhibitor specific for acidocalcisome (Docampo et al., 1995) or 0.3 µM Cyclopiazonic acid, a disruptor of calcium uptake by RE (Furuya et al., 2001) (**Figure 4**). Of note, a store operated Ca2+ channel (SOCE) for calcium influx in *T. equiperdum* and triggered by calcium release from ER was described for the first time in trypanosomatids (Pérez-Gordones et al., 2021) expanding the knowledge on the mechanism of calcium homeostasis in these parasites. Additionally, a membrane tension activated mechanosensitive channel has been described recently in *Trypanosoma cruzi* which, when lacking in knockout parasites, affects calcium regulation and infectivity, among others (Dave et al., 2021). The seeming absence of acidocalcisome involvement is unexpected, since the inositol 1,4,5 triphosphate receptor (IPR3) is localized in acidocalcisomes (Lander et al., 2016), not in the ER, and ablation of the gene coding for the IP3 receptor impairs cell invasion by *T. cruzi* (Chiurillo et al., 2020). However, the proximity between the mitochondrion and acidocalcisome has been demonstrated in *T. cruzi* and *T. brucei* (Miranda et al., 2000; Ramakrishnan et al., 2018), and it has been suggested that the contact of both organelles would facilitate a calcium movement between them (Docampo et al., 2021). Possibly, the unique mitochondrion of the parasite and acidocalcisomes are both operative in regulating the binding to ECM followed by a host cell internalization of the parasite.

Cytoplasmic Ca^2+^ is important for cellular signaling as an ion regulator of many enzymes and proteins activity. The observed decrease in the phosphorylation levels of proteins could be one of the consequences of a calcium uptake from the external milieu in the case of trypomastigotes incubated with ECM, as suggested by the results obtained in the presence of Nifedipine or EGTA, where this decrease is less evident. Additionally, the total phosphatase activity decreases in the presence of Nifedipine or EGTA, almost at the same order of magnitude as Okadaic acid, an inhibitor of the protein phosphatases PP2A and PP1 in *T. cruzi* (Szöör, 2010). Although we did not explore these data further, phosphatases and calcium are associated with distinct biological functions in *T. cruzi*, including host cell invasion (Docampo and Moreno, 1986; Orrego et al., 2014) and differentiation (González et al., 2003). Moreover, decreases in the phosphorylation levels of proteins, including structural proteins, the majority of protein kinases and protein phosphatases, such as protein phosphatase PP1, seem to be a major event in the tissue culture -derived trypomastigotes incubated with the ECM for longer periods of time (2 h), as shown by the phosphoproteomic analysis and modification on enzyme activities, such as hexokinase or pyruvate kinase (Mattos et al., 2019).

In short, we show the uptake of extracellular calcium by *T. cruzi* trypomastigotes by nifedipine-sensitive calcium channel at the very early phase of its interaction with the extracellular matrix and the main role of mitochondria in calcium homeostasis during the interaction process. Calcium uptake is at least partially dependent on the protein composition of the ECM, probably triggering the activation of protein phosphatases, such as PP1 and PP2A, considering that the dephosphorylation of proteins is associated with the invasion process. However, distinct signaling pathways are expected to be triggered, since multiple molecular interactions occur between the parasite and ECM. Our studies thus deepen our understanding of the complexities of host cell invasion by *T. cruzi* trypomastigotes.

## Data Availability Statement

The raw data supporting the conclusions of this article will be made available by the authors, without undue reservation.

## Author Contributions

Conceptualization: MA, WC, and NM. Methodology: NM and GR. Formal analysis: MA, WC, and NM. Resources: MA, WC, NM, and GR. Original draft: NM. Writing review and editing: NM, MA, and WC. Visualization: NM and GR. Supervision: MA and WC. Funding acquisition: MA and WC. All authors contributed to the article and approved the submitted version.

## Funding

The work was supported by grants and fellowships from Fundação de Amparo à Pesquisa do Estado de S. Paulo, FAPESP, Brazil (2014/25494-9, 2017/19854-0 to MA; 2018/03727-2 to NM; 2018/04559-6 to GR), Conselho Nacional de Desenvolvimento Científico e Tecnológico, CNPq, Brazil (304232/2014-9 to MA; 305951/2017-3 to WC; 150980/2017-5 to NM) and Coordenação de Aperfeiçoamento de Pessoal de Nível Superior, CAPES, Brazil. The funders had no role in study design, data collection and analysis, decision to publish, or preparation of the manuscript.

## Conflict of Interest

The authors declare that the research was conducted in the absence of any commercial or financial relationships that could be construed as a potential conflict of interest.

## Publisher’s Note

All claims expressed in this article are solely those of the authors and do not necessarily represent those of their affiliated organizations, or those of the publisher, the editors and the reviewers. Any product that may be evaluated in this article, or claim that may be made by its manufacturer, is not guaranteed or endorsed by the publisher.
