# Supplementary material for: Interaction With the Extracellular Matrix Triggers Calcium Signaling in Trypanosoma cruzi Prior to Cell Invasion
Source: Front Cell Infect Microbiol. 2021 Oct 4;11:731372. doi: 10.3389/fcimb.2021.731372 (PMC8521164; doi:10.3389/fcimb.2021.731372)
Supplement: Supplementary file 1 [file DataSheet_1.pdf]

Supplementary Material

Supplementary Video 1. Fluo-4-AM- loaded trypomastigotes incubated with ECM. Correct loading was verified by fluorescence microscopy before every experiment. The video represents 50 frames of 0.5 s. White bar represents 100 μm.
