# Supplementary material for: Interaction With the Extracellular Matrix Triggers Calcium Signaling in Trypanosoma cruzi Prior to Cell Invasion
Source: Front Cell Infect Microbiol. 2021 Oct 4;11:731372. doi: 10.3389/fcimb.2021.731372 (PMC8521164; doi:10.3389/fcimb.2021.731372)
Supplement: Supplementary file 2 [file DataSheet_3.pdf]

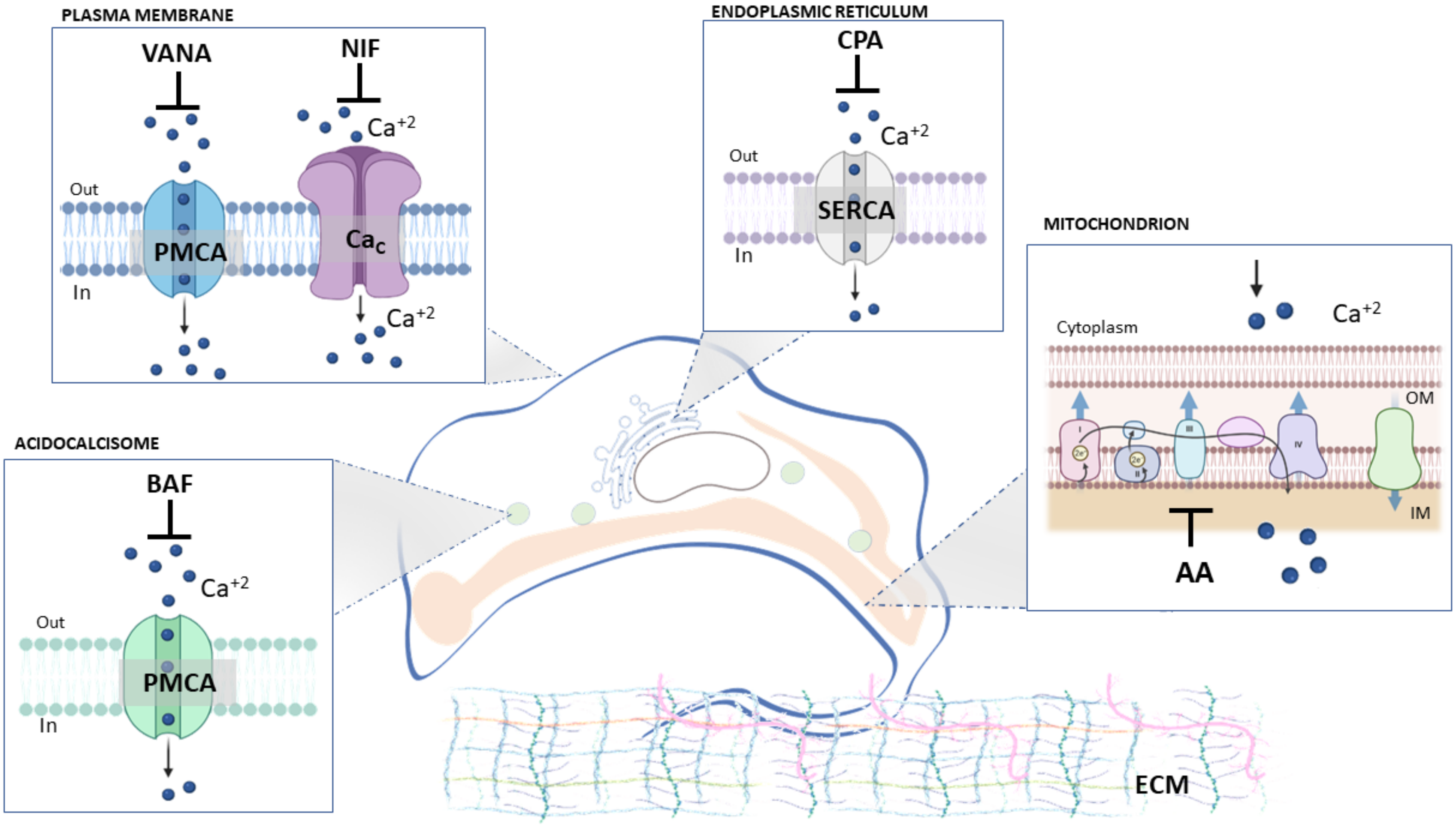

Supplementary Figure 2. Schematic representation of the mechanism of action of the inhibitors employed. VANA (Vanadate), NIF (Nifedipine), PMCA (Plasma Membrane Calcium ATPase), Ca_c (Calcium channel), CPA (Cyclopiazonic Acid), SERCA (sarco/endoplasmic reticulum Ca^{2+}-ATPase), AA (Antimycin A), BAF (Bafilomycin A), ECM (Extracellular Matrix).
