# Supplementary material for: Interaction With the Extracellular Matrix Triggers Calcium Signaling in Trypanosoma cruzi Prior to Cell Invasion
Source: Front Cell Infect Microbiol. 2021 Oct 4;11:731372. doi: 10.3389/fcimb.2021.731372 (PMC8521164; doi:10.3389/fcimb.2021.731372)
Supplement: Supplementary file 3 [file DataSheet_2.pdf]

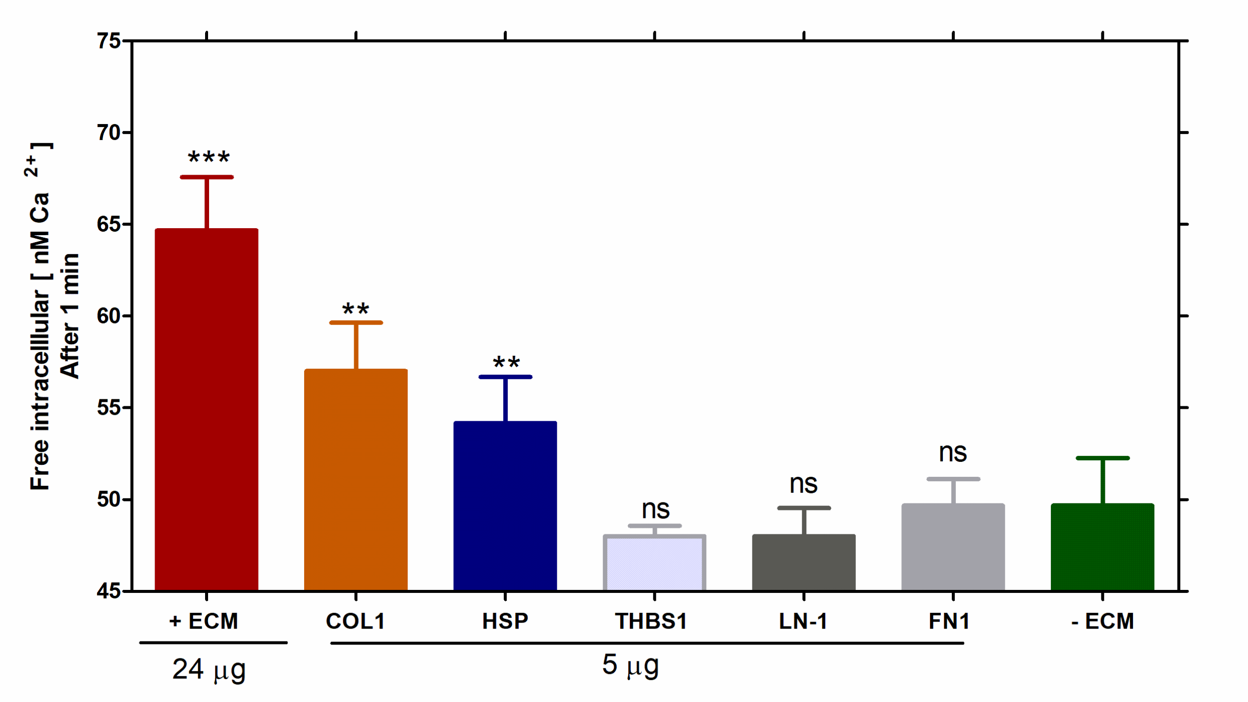

Supplementary Figure 3. Free intracellular calcium concentration in tissue cultured-trypomastigotes in the presence of Collagen 1 (COL1), heparan sulfate (HSP), thrombospondin (THBS1), laminin-111 (LN-1), fibronectin (FN1), ECM or the control without ECM (-ECM).
